# Differences in Characteristics of Peripartum Patients Who Did and Did Not Require an Upgrade to the Intensive Care Unit: A Propensity Score Matching Study

**DOI:** 10.3390/medicina61010163

**Published:** 2025-01-20

**Authors:** Jennifer A. Walker, Jerry M. Yang, Saad Pirzada, Manahel Zahid, Samantha Asuncion, Amanda Tuchler, Gillian Cooper, Allison Lankford, Emad Elsamadicy, Quincy K. Tran

**Affiliations:** 1 Department of Emergency Medicine, Baylor Scott & White All Saints Medical Center, Fort Worth, TX 76104, USA; jennifer.walker@bswhealth.org; 2 Research Associate Program in Emergency Medicine and Critical Care, Department of Emergency Medicine, University of Maryland School of Medicine, Baltimore, MD 21201, USA; jerryyang1223@gmail.com (J.M.Y.); saadpirzada147@gmail.com (S.P.); 3 School of Medicine, University of Baltimore, Baltimore, MD 21201, USA; manahel.zahid@som.umaryland.edu (M.Z.); sasuncion@som.umaryland.edu (S.A.); amanda.tuchler@gmail.com (A.T.); gillian.cooper@som.umaryland.edu (G.C.); 4 Department of Obstetrics, Gynecology and Reproductive Sciences, University of Maryland School of Medicine, Baltimore, MD 21201, USA; allison.lankford@som.umaryland.edu; 5 Division of Maternal Fetal Medicine, Department of Obstetrics and Gynecology, Vanderbilt University Medical Center, Nashville, TN 37232, USA; emad.elsamadicy@vumc.org; 6 Department of Emergency Medicine, University of Maryland School of Medicine, Baltimore, MD 21201, USA; 7 Program in Trauma, The R Adam Cowley Shock Trauma Center, University of Maryland School of Medicine, Baltimore, MD 21201, USA

**Keywords:** critical illness in pregnancy, obstetric complications, peripartum hospitalization, peripartum morbidity, peripartum mortality, peripartum outcomes, peripartum prognostication, peripartum triage, emergency medicine obstetric illness

## Abstract

*Background and Objectives:* This study sought to identify predictors for peripartum patients admitted to non-intensive care wards who later upgraded to the Intensive Care Unit (ICU). *Materials and Methods:* This was a retrospective observational study of patients admitted to the Maternal Fetal Ward between 01/2017 and 12/2022, who later upgraded to the ICU. Upgraded patients were 1:1 propensity score matched with those who remained on the Maternal Fetal Ward (control). The Classification And Regression Tree, a machine learning algorithm, was used to identify significant predictors of ICU upgrade. Multivariable ordinal regression analysis was used to assess the time interval to upgrade. *Results:* From 1855 peripartum patients, we analyzed 37 control and 34 upgrade patients. Mean maternal age (±Standard Deviation) and gestational age for the group was 29.5 (±5.8) years and 31.5 (±7.5) weeks, respectively. The Median Sequential Organ Failure Assessment Score [Interquartile] at ward admission for the controls was 0 [0–1] versus 2 [0–3.3, *p* = 0.001] for upgrade patients. The Sequential Organ Failure Assessment score at Maternal Fetal Ward admission was most predictive, followed by the Acute Physiology and Chronic Health Evaluation II (APACHE II) score, and lactate dehydrogenase levels. The APACHE II score was also associated with ICU upgrade within 12 h of hospital admission (OR 1.4, 95% CI 1.08–1.91, *p* = 0.01). *Conclusions:* Compared to control patients, peripartum patients upgraded to the ICU are associated with higher physiologic scores at Maternal Fetal Ward admission. Until further studies are performed to confirm our observation, clinicians should pay attention to these physiologic scores, since they may be associated with higher-risk patients.

## 1. Introduction

When peripartum patients are hemodynamically unstable or hemorrhaging, admission directly to an Intensive Care Unit (ICU) rather than to the Maternal Fetal Ward (MFW) can be an obvious clinical decision. Well-appearing patients admitted to the MFW, however, occasionally become unstable, requiring transfer to the ICU. In the non-obstetric population, studies illustrate that patients requiring transfer from the ward to the ICU have increased morbidity and mortality [1,2,3]. Continued concerns regarding the maternal mortality rate exist; hence, it is crucial to understand how to readily recognize peripartum patients at admission with risk for decompensation [4].

Because early recognition of critical illness is associated with improved outcomes, work has been conducted regarding early warning scores (EWSs) in admitted patients [5,6]. Similar work has occurred in the maternal population, where there is some promise that maternal early warning scores (MEWSs) during admission can identify clinically declining inpatients [7,8]. The EWS and MEWS are used to identify declining inpatients, however, rather than prior to admission.

Other studies to identify high-risk obstetric populations at admission exist. These have sought to identify higher-risk demographic or socioeconomic groups, identifying medical and social risk factors associated with obstetric ICU admissions as well as describing common diagnoses at ICU admission [9,10,11,12,13,14]. Other scores, such as Acute Physiology and Chronic Health Evaluation (APACHE) scores and Sequential Organ Failure Assessment (SOFA) scores have been assessed mostly for predicting maternal mortality but not for predicting risk at admission of clinical decline [15,16]. Furthermore, almost all of these studies did not include a control group of patients to compare with those patients who were admitted to the ICU.

The Sepsis in Obstetrics Score (SOS) has been validated to predict ICU needs in perinatal patients, but this score does not apply to admissions for other causes [17].

Our study seeks to identify clinical indicators and physiologic scores on admission that differ between peripartum patients requiring versus not requiring ICU upgrade. A machine learning algorithm using a Classification And Regression Tree (CART) was used to identify predictors associated with ICU upgrade. Since we anticipated smaller sample sizes in this study due to this specific patient population, the use of the CART model is more advantageous than the traditional multivariable logistic regression, as the CART logarithm can handle datasets with missing data without having to impute or drop cases like the multivariable logistic regression [18]. Furthermore, the CART algorithm is not as restricted to the number of outcome variables, as the traditional multivariable logistic regression requires a certain number of outcome variables for each number of independent variables.

Interventions occurring upon transfer to the ICU, such as intubation and mechanical ventilation, were evaluated, as was the time to upgrade. In defining these predictors, it may be possible to identify the need for critical care services earlier in peripartum admission, thereby improving outcomes.

## 2. Materials and Methods

This is a retrospective observational study evaluating all peripartum patients admitted to an MFW at a quaternary care center between 1 January 2017 and 31 December 2022. At the study institution, there is an Obstetric and Maternal Fetal Medicine (OMF) service available 24/7 to provide care for peripartum patients. When a peripartum patient needs admission, the OMF team triages and accepts appropriate patients to the MFW. If a patient is admitted to the MFW, and there is concern that a patient clinically declines, the OMF team and ICU physicians coordinate the level of care. The OMF team at the study institution includes a critical care trained obstetric physician.

Peripartum patients admitted to the MFW from the study institution’s Emergency Department or another hospital’s non-ICU setting who were greater than 20 weeks gestation or less than four weeks postpartum were included. Patients transferred from outside ICUs or admitted directly to the ICU were excluded. Also excluded were patients with a gestational age less than 20 weeks or a diagnosis of abnormal pregnancy. The control group included patients who were admitted to the MFW unit but did not transfer to the ICU. This study was IRB-approved with a waiver of consent at the senior author’s institution (HP-00084554).

The primary outcome for this study was the difference between physiologic scores at admission in the control versus the ICU upgrade groups. Secondary outcomes included predictors for ICU upgrade and time intervals from MFW admission to ICU upgrade. Mortality was not an outcome because a previous study showed that the mortality rate for peripartum patients at this institution was too low to perform a sufficient regression analysis [19].

Electronic medical records were utilized to collect patients’ demographic information, maternal comorbidities, clinical information at the time of ICU upgrade, and interventions in the ICU after upgrade. All data were extracted into a standardized Excel spreadsheet (Microsoft Corp, Redmond, Washington, DC, USA). Prior to commencement of data collection, investigators were trained by a senior investigator using sets of 5 sample charts, until data accuracy between investigators reached 90%.

Clinical data were collected to calculate physiologic scores, including the SOFA score, APACHE II score, and shock index. The Respiratory Rate-Oxygenation (ROx) index, which denotes a patient’s work of breathing and likelihood of requiring invasive mechanical ventilation, was also calculated. Laboratory values including the serum lactate level, white blood cell (WBC) count, liver function, lactate dehydrogenase (LDH), uric acid, and international normalized ratio (INR) were collected. The time interval from admission to the MFW and the time to decision of ICU upgrade were obtained. Any missing data for physiologic scores or laboratory values were imputed as normal values. After we re-evaluated the patients with missing data, most of the missing data were the follow-up from initial, normal laboratory evaluations. Therefore, their repeats were deemed not clinically indicated by clinicians.

In this exploratory study, a sample size analysis was not performed due to the retrospective nature of this study and anticipated small population. All eligible patients during the time period were included. Descriptive analyses were performed to compare the data between groups. Continuous data were first examined for their distribution via a histogram and are reported as the mean (±Standard Deviation [SD]) or median [Interquartile (IQR)]. Categorical data are expressed as percentages. Variables were analyzed with Student’s *t*-test, the Mann–Whitney U test, or Chi-squared tests as appropriate. The results from these analyses are reported as the difference between groups and the 95% Confidence Interval (95% CI) of the differences, with control patients as the reference.

In order to match the control and ICU upgrade patients, propensity score matching was performed. Multivariable logistic regression using a priori defined variables (Appendix A.1) for the need of ICU upgrade was constructed to calculate the propensity score for each MFW patient. An optimal algorithm with 1:1 matching of the propensity score without replacement was carried out, using a strict caliper of 0.1 instead of the recommended 0.2 caliper to ensure that individuals in the group were matched, since most of our matching criteria were categories and these categories are not as strict [20]. Therefore, we opted for a stricter caliper so that our patient population could be more balanced. Information about the propensity score for matching patients is provided in Appendix A.2.

To identify predictors of ICU upgrade, we used the Classification And Regression Tree (CART). The variables to be included in the CART were determined a priori (Appendix B). CART is a machine learning algorithm that utilizes recursive partitioning to identify a series of dichotomous splits (ICU upgrade versus no ICU upgrade). The CART algorithm examined the interactions between the variables to provide the most sensitive and specific splits. The series ends at a “terminal node” with a final branch point signifying the outcome of interest (i.e., no further split is possible). The CART model then assigns the most important classification a “relative variable importance (RVI)” of 100%. Other variables are assigned subsequent to RVI, as a percentage of the most important factor. The RVI does not indicate the order of predictors’ appearance in the tree diagram, which demonstrates how predictors interact among themselves. The CART model was performed with 10-fold cross-validation, a minimum count of 3 patients for each node, and the depth of the tree of 20. Performance of the CART model was assessed via the Area Under the Receiver Operating Curve (AUROC) and the misclassification cost. The AUROC indicated the discriminatory capability of the CART model. A model with an AUROC value approaching 1.0 is considered to have perfect discriminatory capability. Higher misclassification cost would suggest that the CART model has a higher risk of producing prediction errors. A sensitivity analysis was performed using CART analysis and the dataset without imputation of the missing laboratory values to assess the implication of the imputation of the missing data (Appendix C.1 and Appendix C.2). The setting of the CART model for the sensitivity analysis was the same as the main model.

We also performed a multivariable ordinal logistic regression to assess independent variables associated with patients’ time interval from arrival to the MFM ward to the time of ICU upgrade. The time interval was ranked according to the distributions from examining its histogram. For multivariable ordinal logistic regression, the time interval from arrival at the MFW and to ICU upgrade was ranked from 0 (0–12 h), 1 (12.1–24.0 h), and 2 (>24.0 h). The results from the multivariable ordinal logistic regression are expressed with a correlation coefficient, *p*-value, and 95% CI. A positive correlation coefficient is most associated with the lowest rank (rank of 0, or time to ICU upgrade <12 h), while a negative correlation coefficient is most associated with the highest rank (rank of 2, or time to ICU upgrade >24 h). Goodness of fit for the ordinal logistic regression was assessed by the Sommer’s D and Goodman–Kruskal Gamma tests. Goodness of fit increases when the values of these tests approach 1.0. The list of variables and the results for the ordinal logistic regression are provided in Appendix B and Appendix D.

The propensity score matching was performed with XLSTAT [21]. Descriptive analyses, CART, probit logit regression, and multivariable ordinal logistic regression were performed with minitab version 19 [22]. All statistical analyses with a *p*-value < 0.05 were considered statistically significant.

## 3. Results

**Figure 1:**
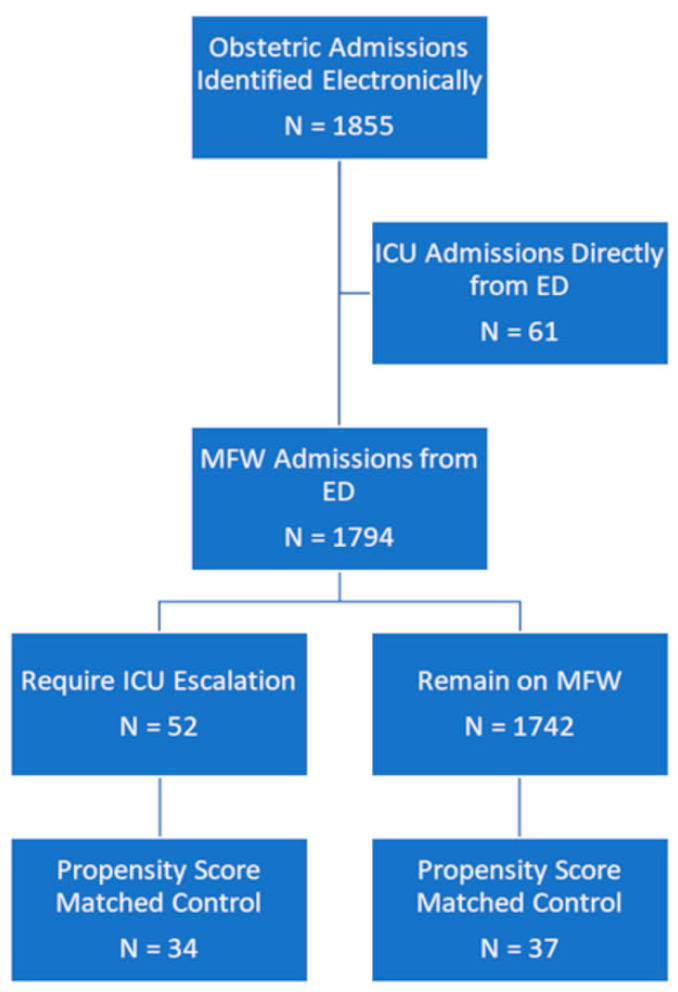
Flow diagram of patient selection illustrating source of study population. ICU = Intensive Care Unit; ED = Emergency Department; MFW = Maternal Fetal Ward.

There were a total of 1855 peripartum patients who were admitted to the MFW. The number that remained on the MFW was 1742 (94%), while 52 (3%) were upgraded to the ICU (Figure 1). The 1:1 propensity score matching resulted in 37 control patients remaining on the MFW and 34 patients upgraded to the ICU (Figure 1). The mean age (±Standard Deviation [SD]) for patients was similar between the control group (29.4 years ± 5.6) and ICU-upgraded group (29.5 years ± 6.1, *p* = 0.96) (Table 1). All other demographic information between groups was non-statistically similar (Table 1). The diagnoses at ICU admission are presented in Table 2.

Patients who required ICU upgrade were associated with statistically higher median (Interquartile Range [IQR]) physiologic scores than control patients. The median SOFA score upon arrival to the hospital for control patients was (0 [0–1]), compared with ICU-upgraded patients (2 [0–3.3], *p* = 0.001). The mean (±SD) of APACHE II upon admission was lower for control patients (3.1 ± 2.4) than ICU-upgraded patients (5.2 ± 3.3, *p* = 0.004) (Table 3). The effect sizes between the groups were already significant at the current sample size; therefore, post hoc power analysis was not performed.

Patients who upgraded to the ICU were associated with a statistically longer mean (±SD) hospital length of stay (HLOS), compared to control patients—HLOS 9 days (95% CI −14.3, −3.6, *p* = 0.002). One patient (3%) from the ICU-upgraded group died, while none from the control group died (Table 3). All survivors were discharged home directly from the hospital.

The most common ICU interventions performed for patients in the ICU cohort were vasopressors (32.4%), mechanical ventilation (23.5%), and any transfusion given (20.6%). Four (10.8%) patients in the control group were placed on a high-flow nasal cannula and one patient underwent Cesarean section. There were no other ICU interventions performed on the 37 control group patients (Table 4).

### 3.1. Classification and Regression Tree Analysis for ICU Upgrade Prediction

**Figure 2:**
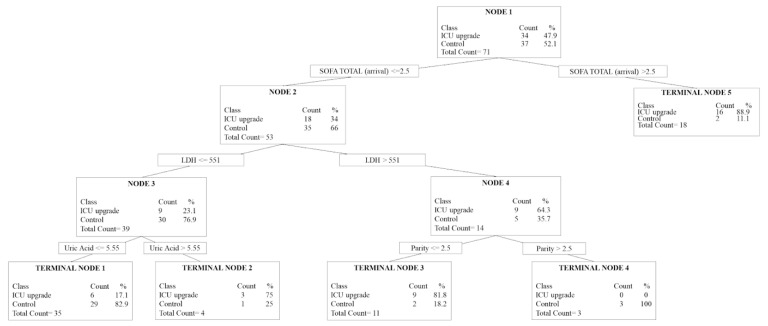
The tree diagram from the Classification And Regression Tree analysis. In this model, missing laboratory values were imputed as normal values. The Area Under Receiver Operating Curve was 0.67 (95% CI 0.54–0.80) with a misclassification cost = 0.57. The CART algorithm examined the interactions between the variables to provide the split (node) with the highest sensitivity and specificity. When the interaction cannot produce any further split, i.e., the variable may achieve the highest possible association with the outcome of interest, the series ends at a “terminal node”. ICU = Intensive Care Unit; LDH = Lactate dehydrogenase; SOFA = Sequential Organ Failure Assessment.

**Figure 3:**
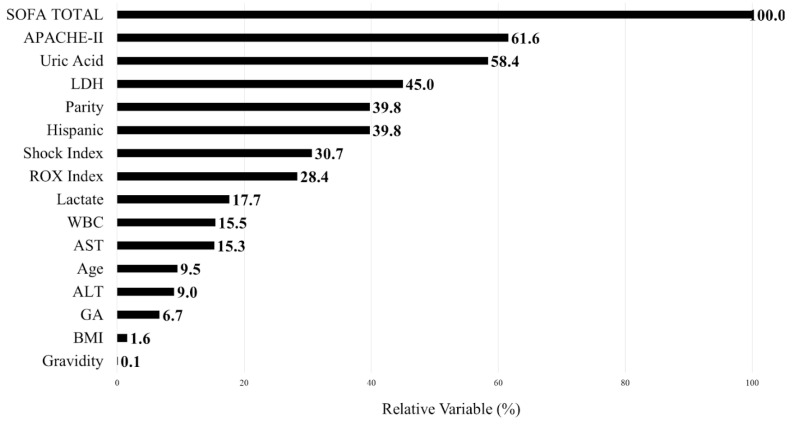
Relative variable importance of clinical variables predicting the need for Intensive Care Unit upgrade after admission to the Maternal Fetal Ward. Variable importance measures model improvement when splits are made on a predictor. Relative importance is defined as a % improvement with respect to the top predictor. AST = Aspartate aminotransferase; ALT = Alanine aminotransferase; APACHE II = Acute Physiology and Chronic Health Evaluation II; BMI = Body mass index; GA = Gestational age; LDH = Lactate dehydrogenase; Rox = Respiratory Rate-Oxygenation index; SOFA = Sequential Organ Failure Assessment; WBC = White blood cell.

The CART analysis suggested that a SOFA score of >2.5 upon admission was an important predictor for ICU upgrade, as 88.9% of ICU-upgraded patients had a SOFA score >2.5 (Figure 2, Terminal Node 5). Similarly, SOFA at arrival was considered the most important factor predicting the need for ICU upgrade (RVI 100%) (Figure 3). For patients with a SOFA score upon admission ≤2.5, serum LDH of 551 (U/L) was another cut-off value for the prediction of patients’ need for ICU upgrade (Figure 2, Node 2). When patients had an LDH ≤551 and uric acid ≤5.5 mg/dL, approximately 83% (29/35) of patients were not upgraded to the ICU (Figure 2, Terminal node 1). On the other hand, when patients had an LDH >551 (Figure 2, Node 4), patients’ parity became important. Patients with multiple parity (>3) were less likely to be upgraded to ICU (Figure 2, Terminal Node 4), while patients with parity <3 were more likely to be upgraded to the ICU (Figure 2, Terminal Node 3).

Besides the admission SOFA score, the other top five predictive variables that were identified by the CART included APACHE II (RVI 61.6%), uric acid (RVI 58.4%), LDH, (RVI 45%) and parity (RVI 39.8%) (Figure 3). The AUROC for this CART model was 0.67 (0.54–0.80), and the misclassification cost was 0.57.

The middle-ranked variables such as the shock index (RVI 30.7%), ROx index (RVI 28.4%, lactate (RVI 17.7%), and WBC (RVI 15.5%) may be clinically intuitive, as they may be considered toward the decision to upgrade patients to the ICU. The clinical implications for the lower-ranked variables with RVI <10% might not be clear, as some authors might consider factors with RVI <10% as not clinically significant.

Sensitivity analysis using the CART model without imputation of the missing laboratory values suggested that the SOFA, APACHE II, and LDH scores at arrival at the MFW remained the top five important variables (Appendix C.2). The CART model without imputation of missing laboratory values had a similar AUROC as the first model, but with a higher misclassification cost.

### 3.2. Ordinal Logistic Regression Results—Time to ICU Upgrade

An ordinal logistic regression model was calculated to identify factors associated with the time interval between MFW admission and upgrade to the ICU. Chronic hypertension (coefficient 0.36 [1.08–1.91], *p* = 0.01), tobacco use (coefficient 2.65 [1.69–119.38], *p* = 0.02), and a higher APACHE-II score at hospital admission (coefficient 3.16 [1.29–427.3], *p* = 0.03) were associated with a shorter time to ICU upgrade (Table 5, full ordinal regression results found in Appendix D).

## 4. Discussion

Efforts to reduce maternal mortality have included studies that determined indicators of peripartum risk during admission such as Maternal Early Warning Scores (MEWS). Socioeconomic and medical comorbidities are also known to indicate high-risk admissions, and Sepsis in Obstetrics Score (SOS) has been validated to indicate risk for ICU admission in septic peripartum patients. We are not aware of any studies that exist looking at clinical scores identifying high-risk admissions in the general peripartum population. Our study identified several predictors available at admission that were associated with the need for ICU upgrade. The SOFA score at admission was the most important physiologic score associated with ICU upgrade. Other variables predicting ICU upgrade included the APACHE II, uric acid, LDH, and parity levels. It took less than 12 h for patients with higher APACHE II scores to upgrade.

Currently, calculation of an admission SOFA score is not a typical practice for peripartum patients, but the use of a SOFA score in peripartum patients at ICU admission is associated with prognosis [16]. This study suggests that using a SOFA score at hospital admission might improve the triage of peripartum patients to appropriate levels of care.

This study sought to identify clinical characteristics that were present both on admission and after admission to the MFW in contrast to previous studies that looked at demographic and clinical variables prompting transfer or admission to the ICU [9,10,11,12,13,14]. Furthermore, previous studies have not demonstrated the change in physiologic scores between MFW admission and ICU transfer. Because this study accounts for changes in physiologic and laboratory values available upon admission, it provides additional evidence to aid clinicians in their decision-making process.

This study also identified a history of essential hypertension and smoking tobacco as two predictors for a short interval to ICU upgrade. Having a history of essential hypertension and its association with a quick upgrade to an ICU is clinically intuitive as patients with history of hypertension are at higher risk for developing pre-eclampsia or eclampsia [23]. On the other hand, peripartum patients with a history of smoking were also associated with higher risks for placenta previa, placental abruption, ectopic pregnancy, and premature rupture of the membrane [24]. Thus, smoking predisposed peripartum patients to have a high risk of ICU upgrade due to bleeding or sepsis. However, since finding the underlying mechanisms to explain this result was outside the scope of this study, further studies with larger sample sizes are necessary to confirm or refute our findings.

Unexpected transfers to the ICU are associated with worse outcomes [1,2,3] as is a delay of transfer due to a lack of ICU beds [25]. Recognizing and identifying variables at admission can allow for improved triage of patients at high risk of deterioration.

This study contained well-matched control and ICU-upgraded patients to compare physiologic scores that may have indicated future decline. Most studies looking at critically ill peripartum patients do not feature matched controls. While these patients were well matched by age, parity, and diagnosis at admission, a limitation of this study is that other comorbidities were unaccounted. Many demographic variables, however, had similarities between the control and study groups. Another limitation was the retrospective nature of the data as well as the small sample size, yet, even with a small sample size, the major physiologic scores upon arrival were statistically significant. Thus, since there was already statistical significance between all physiologic scores, we did not perform post hoc power analysis, which is usually only performed when there is no statistical significance between outcomes. The small sample size did cause a few independent variables, including the LDH, to have wide 95% CI intervals. Furthermore, the CART’s AUROC was only moderate, most likely because of the small sample size and homogenous patient population. Future studies should involve a large sample size, multi-center and more heterogenous patients, and stricter propensity score matching. A large sample size also affords better pruning and increased specificity of the model by allowing a larger minimum number of patients for each node or terminal node. Such ideal studies will provide more definitive recommendations for the criteria used for upgrading peripartum patients. Lastly, this study may not be generalizable since the acuity and management at a single, quaternary center with a critical care trained obstetric team may differ compared to other settings.

This is an area that deserves further research, as it may reduce the morbidity associated with in-hospital clinical decline and ICU transfers. Specifically, studies with larger sample sizes and different hospital settings will aid in validating our model and specific admission physiologic scores as well as other variables indicating potential clinical decompensation. Additionally, looking at other clinical scores that are more easily calculated at admission, such as the qSOFA, may be beneficial. Furthermore, using automated software to automatically calculate SOFA and APACHE II scores has been reported to be feasible and have good discriminatory value for in-hospital mortality and ICU length of stay [26]. This automated software reduces bias and human errors by minimizing clinicians’ data inputs. Therefore, using automated software to calculate physiologic scores, besides reducing clinicians’ workload, could help with resource allocation and triaging who should be admitted directly to an ICU bed, especially when ICU bed availability is low, or help better triage high-risk patients such as peripartum patients. Similar to the SOFA and APACHE II scores, the Modified Obstetric Early Warning Score (MOEWS) has been tested to compare its utility with the APACHE II score [27]. In a retrospective study involving 352 pregnant women, the MOEWS score was associated with a higher AUROC than the APACHE II score for predicting severe obstetric complications such as pre-eclampsia, shock, and pulmonary embolism. However, the MOEWS score has not been studied as extensively as the SOFA and APACHE II scores in the peripartum population. Therefore, further studies are needed to validate the MOEWS score for peripartum patients. In contrast, the simpler physiologic score qSOFA was a poor tool for predicting maternal comorbidity in a retrospective study of 104 pregnant patients [28].

## 5. Conclusions

This study illustrated that patients requiring ICU upgrade compared to the control group were associated with higher physiologic scores at admission to the MFW. ICU-upgraded patients also demonstrated a larger change in their physiologic scores within 24 h of admission to the MFW. Although further studies are needed to confirm this observation, clinicians could consider using SOFA and APACHE II scores as triage tools to determine who may need direct admission to the ICU.

## Figures and Tables

**Table 1:** Baseline characteristics of control patients and patients who required Intensive Care Unit upgrade.

Variable	All Patients	Control	ICU Upgrade	Difference Between Groups	95% CI	*p*
	N = 71	N = 37	N = 34	NA	NA	
Age (years), mean (SD)	29.5 (5.8)	29.4 (5.6)	29.5 (6.1)	−0.1	(−2.88, 2.69)	0.96
Body mass index, mean (SD)	31.5 (8.7)	32.1 (9.1)	30.8 (8.4)	1.3	(−2.82, 5.42)	0.53
Gestational age (week), mean (SD)	31.2 (7.5)	31.8 (7.5)	30.5 (7.5)	1.4	(−2.19, 4.92)	0.45
Gravidity, median [IQR1–IQR3]	3.0 [2.0–5.0]	3.0 [2.0–5.0]	3.0 [1.8–4.3]	0.0	(−1.00, 1.00)	0.64
Parity, median [IQR1–IQR3]	1.0 [0.0–2.0]	2.0 [1.0–2.5]	1.0 [0.0–2.0]	0.0	(0.00, 1.00)	0.18
Race, N (%)						
Asian	1 (2.9)	0 (0.0)	1 (2.9)	−0.03	(−0.09, −0.03)	0.48
Black	30 (42.2)	17 (46.0)	13 (38.2)	0.08	(−0.15, 0.31)	0.63
Hispanic	18 (25.3)	11 (29.7)	7 (20.6)	0.09	(−0.11, 0.29)	0.42
White	22 (31.0)	9 (24.3)	13 (38.2)	−0.14	(−0.35, 0.07)	0.3
Marital status, N (%)						
Divorced	2 (2.8)	1 (2.7)	1 (2.9)	−0.002	(−0.08, 0.07)	0.99
Single	41 (57.8)	21 (56.8)	20 (58.8)	−0.02	(−0.25, 0.21)	0.99
Married	28 (39.4)	15 (40.5)	13 (38.2)	0.02	(−0.20, 0.25)	0.99
Past medical history, N (%)						
Hypertension	15 (21.1)	6 (16.2)	9 (26.5)	−0.15	(−0.43, 0.13)	0.29
Diabetes mellitus	8 (11.3)	4 (11.8)	4 (10.8)	−0.01	(−0.16, 0.14)	0.99
Chronic kidney disease	2 (2.8)	0 (0)	2 (5.9)	−0.06	(−0.14, 0.02)	0.23
Asthma	15 (21.1)	5 (13.5)	10 (29.4)	−0.16	(−0.35, 0.03)	0.15
Any cardiac disease	2	2 (5.4)	0 (0)	0.05	(−0.02, 0.13)	0.49
Social history, N (%)						
Tobacco	9 (12.7)	4 (10.8)	5 (14.7)	−0.04	(−0.19, 0.12)	0.73
IVDU	4 (5.6)	0 (0.0)	4 (11.8)	−0.12	(−0.23, −0.01)	0.05
Psychiatric history—anxiety/depression, N (%)	13 (18.3)	5 (13.5)	8 (23.5)	−0.1	(−0.28, 0.08)	0.36
Any obstetric/gynecologic history, N (%)	22 (31.0)	11 (29.7)	11 (33.3)	−0.04	(−0.25, 0.18)	0.8

Abbreviations: ICU = Intensive Care Unit; SD = Standard Deviation; IQR = Interquartile Range; N = Number of patients; IVDU = Intravenous drug use.

**Table 2:** Diagnoses of peripartum patients who were upgraded to the Intensive Care Unit, after being admitted first to the Maternal Fetal Ward.

Diagnosis at ICU Admission, N (%)	All Patients (N = 34)	Antepartum (N = 16)	Postpartum (N = 18)
Acute respiratory failure	17 (50.0)	13 (81.3)	4 (22.2)
Cardiomyopathy	4 (11.8)	1 (6.3)	3 (16.7)
COVID-19	11 (32.4)	11 (68.8)	0 (0.0)
Hemorrhage	3 (8.8)	0 (0.0)	3 (16.7)
Pre-eclampsia/Eclampsia	8 (23.5)	1 (6.3)	7 (38.9)
Sepsis	2 (5.9)	1 (6.3)	1 (5.6)

COVID-19 = coronavirus disease of 2019; ICU = Intensive Care Unit.

**Table 3:** Comparison of clinical information and hospital outcomes between control patients and those who required Intensive Care Unit upgrade.

Variable	All Patients	Control	ICU Upgrade	Difference Between Groups	95% CI	*p*
	N = 71	N = 37	N = 34	NA	NA	
Arrival SOFA score, median [IQR1–IQR3]	0.0 [0.0–3.0]	0.0 [0.0–1.0]	2.0 [0.0–3.3]	−2	(−3.0, −0.0)	0.001
24 h SOFA score, median [IQR]	2.0 [0.0–3.0]	1.0 [0.0–2.0]	3.0 [0.0–6.0]	−2	(−3.0, −1.0)	0.001
Change in SOFA score, median [IQR1–IQR3]	1.0 [0.0–2.0]	0.0 [0.0–1.0]	1.0 [0.0–3.0]	0	(−1.0, −0.0)	0.150
Arrival APACHE II, mean (SD)	4.1 (3.03)	3.1 (2.4)	5.2 (3.3)	−2.1	(−3.45, −0.68)	0.004
24 h APACHE II, mean (SD)	4.3 (2.8)	3.1 (2.6)	5.7 (2.4)	−2.7	(−3.86, −1.50)	0.001
Change in APACHE score, mean (SD)	7.9 (7.5)	2.8 (2.1)	13.5 (7.2)	−10.7	(−13.33, −8.11)	0.001
Arrival shock Index, mean (SD)	0.8 (0.2)	0.8 (0.2)	0.8 (0.2)	0	(−0.17, 0.03)	0.175
24 h shock index, mean (SD)	0.8 (0.2)	0.8 (0.2)	0.8 (0.2)	0	(−0.07, 0.08)	0.850
Change in shock index, mean (SD)	11.6 (11.3)	21.8 (5.4)	0.6 (0.3)	21.2	(19.36, 22.98)	0.001
Arrival ROx index, mean (SD)	20. 6 (7.0)	22.5 (5.4)	18.5 (7.9)	4.1	(0.85, 7.32)	0.014
24-h ROx index, mean (SD)	20.5 (8.3)	22.3 (7.5)	18.5 (8.9)	3.8	(−0.10, 7.72)	0.056
Change in ROx index, median [IQR1–IQR3]	20.2 [12.5–32.3]	0.0 [0.0–1.0]	13.6 [7.05–19.8]	−13.1	(−16.65, −9.85)	0.001
WBC (counts/uL), mean (SD)	10.9 (4.9)	10.1 (4.0)	11.7 (5.7)	−1.6	(−3.98, 0.75)	0.177
Lactate (mmol/L)—Admission, median, [IQR]	1.4 [0.9–2.0]	1.1 [1.0–1.9]	1.4 [0.9–2.0]	−0.1	(−0.6, 0.4)	0.721
Lactate (mmol/L)—24 h/Upgrade, median, [IQR]	1.4 [0.9–2.2]	1.2 [0.9–1.8]	1.5 [0.8–2.6]	−0.3	(−1.3, 0.4)	0.544
AST (U/L)—Admission, median, [IQR]	42.0 [22.0–64.0]	35.0 [21.0–72.0]	44.0 [26.5–63.3]	−6.0	(−21, 7)	0.335
AST (U/L)—24 h/Upgrade, median, [IQR]	37.0 [25.0–62.5]	32.0 [23.0–48.5]	45.0 [27.0–67.0]	−7.0	(−20, 3)	0.152
ALT (U/L)—Admission, median, [IQR]	26.0 [18.0–58.8]	26.0 [15.0–73.5]	26.0 [22.0–41.0]	−3	(−10, 8)	0.568
ALT (U/L)—24 h/Upgrade, median, [IQR]	27.5 [17.5–43.0]	25.0 [15.5–72.3]	29.0 [20.0 = 41.3]	−0.5	(−10, 12)	0.923
LDH (U/L)—Admission *, mean (SD)	812 (840)	587 (527)	1016 (1017)	−429	(−920, 62)	0.085
Uric acid (mg/dl)—Admission *, mean (SD)	5.6 (1.7)	5.2 (1.4)	6.2 (1.9)	−1.0	(−2.2, 0.2)	0.113
Intervals from arrival to ICU upgrade (hours), median [IQR1–IQR3]	NA	NA	18.71 [9.9, 39.6]	NA	NA	NA
ICU length of stay (days), median [IQR1–IQR3]	NA	NA	3.5 [2.2, 6.4]	NA	NA	NA
Hospital length of stay (days), mean (SD)	8.0 (11.45)	3.7 (2.8)	12.7 (15.1)	−8.92	(−14.3, −3.6)	0.002
Hospital disposition, N (%)						
Dead, N (%)	1 (1.4)	0 (0)	1 (2.9)	−0.03	(−0.09, 0.03)	0.479
Discharge home directly, N (%)	70 (98.6)	37 (100)	33 (97.1)	0.03	(−0.03, 0.09)	0.480
Rehab, N (%)	NA	NA	NA	NA	NA	NA

Abbreviations: ICU = Intensive Care Unit; SOFA = Sequential Organ Failure Assessment; IQR = Interquartile Range; APACHE-II = Acute Physiology and Chronic Health Evaluation; SD = Standard Deviation; ROx index = Respiratory Rate-Oxygenation index, WBC = White blood cell; AST = Aspartate aminotransferase; ALT = Alanine aminotransferase; LDH = Lactate dehydrogenase; INR = International normalized ratio; N = Number of patients. * ICU upgrade values not available.

**Table 4:** Comparisons of interventions during hospital stay between control patients and those who required Intensive Care Unit upgrade.

Variable	All Patients	Control	ICU Upgrade	Difference Between Groups	95% CI	*p*
	N = 71	N = 37	N = 34	NA	NA	
Mechanical ventilation, N (%)	12 (16.9)	4 (10.8) *	8 (23.5)	−0.13	(−0.30, 0.05)	0.21
Vasopressor, N (%)	11 (15.5)	0 (0)	11 (32.4)	−0.32	(−0.48, −0.17)	0.001
Nicardipine infusion, N (%)	4 (5.6)	0 (0)	4 (11.8)	−0.12	(−0.23, −0.01)	0.05
Any transfusion, N (%)	7 (9.9)	0 (0)	7 (20.6)	−0.21	(−0.34, −0.07)	0.004
Massive Transfusion Protocol (total transfusion ≥10 units), N (%)	1 (1.4)	0 (0)	1 (2.9)	−0.03	(−0.09, 0.03)	0.48
CRRT, N (%)	3 (4.2)	0 (0)	3 (8.8)	−0.09	(−0.18, 0.01)	0.11
Mechanical support (ECMO OR balloon pump)	4 (5.6)	0 (0)	4 (11.8)	−0.12	(−0.23, −0.01)	0.05
Type of OBGYN intervention, N (%)						
Cesarean section	5 (7.0)	1 (0.27)	4 (0.1)	−0.09	(−0.21, 0.03)	0.12
D&C or D&E	3 (4.2)	0 (0)	3 (0.1)	−0.09	(−0.18, 0.01)	0.11
Delivery (induction OR vaginal delivery)	2 (2.8)	0 (0)	2 (0.1)	−0.06	(−0.14, 0.02)	0.23

Abbreviations: N = Number of patients; CRRT = Continuous renal replacement therapy; ECMO = Extracorporeal membrane oxygenation; OBGYN = Obstetrics and gynecology; D&C = Dilation and curettage; D&E = Dilation and evacuation. * These patients received a high-flow nasal cannula and remained in the Maternal Fetal Ward for further observation.

**Table 5:** Results of ordinal logistic regression to measure the association between the patient characteristics and time interval between Maternal Fetal Ward admission and Intensive Care Unit upgrade (significant variables only).

Variable	Coefficient	OR	95% CI	*p*
Hospital admission APACHE-II	0.36	1.44	1.08–1.91	0.01
Past medical history of HTN	2.65	14.19	1.69–119.38	0.02
History tobacco use	3.16	23.47	1.29–427.3	0.03

Sommer’s D = 0.65; Goodman–Kruskal Gamma = 0.65; Kendall’s Tau-a = 0.43. Abbreviations: APACHE-II = Acute Physiology and Chronic Health Evaluation II; HTN = Hypertension.

**Figure A1:**
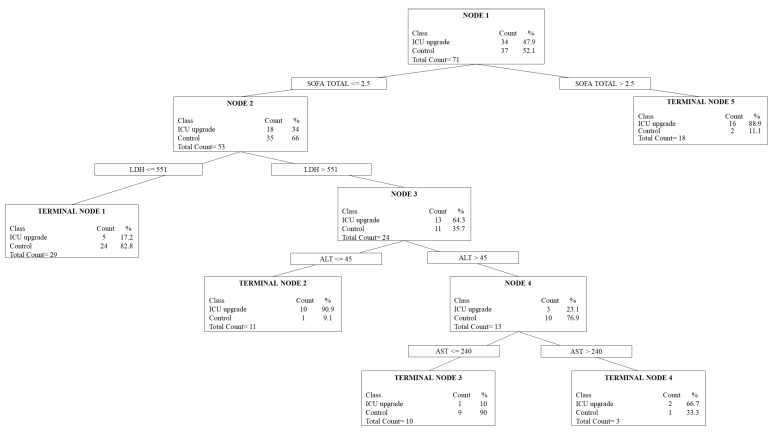
Tree diagram of the Classification and Regression Tree (CART).

**Figure A2:**
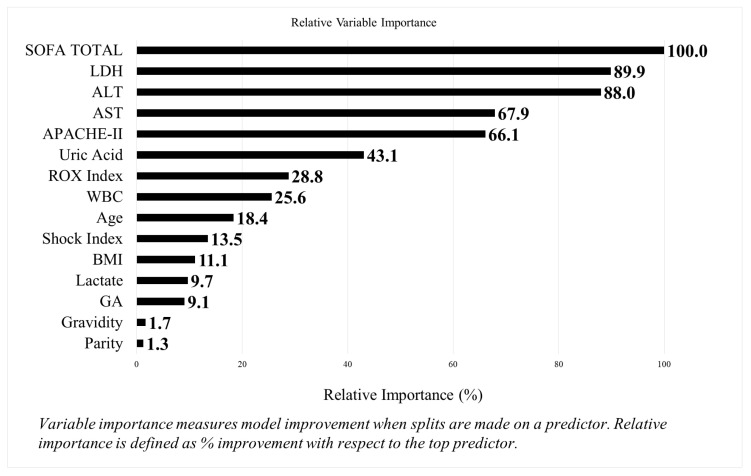
Relative variable importance of the Classification and Regression Tree (CART).

## Data Availability

No new data were created or analyzed in this study.

## Appendix A. Results from the Propensity Score Matching for the Needs of Upgrade to an Intensive Care Unit

### Appendix A.1. Variables for Propensity Score Matching for the Needs of Upgrade to an Intensive Care Unit

**Table A1:** Variables for propensity score matching for the needs of upgrade to an intensive care unit.

Variable
Age
Antepartum at transfer
Postpartum at tramsfer
Diagnosis at transfer
Preeclampsia
Bleeding/Hemorrhage
Infection/Sepsis
Cardiac Disease/Tachycardia
Hypoxia/Respiratory Distress

### Appendix A.2. Propensity Score for Patients Being Included in the 1:1 Propensity Score Matching Between Patients Who Required Upgrade to an Intensive Care Unit Versus Control Patients

**Table A2:** Propensity score for patients being included in the 1:1 Propensity Score Matching between patients who required upgrade to an Intensive Care Unit versus control patients.

ICU Upgrade	Logit (Propensity Score)	Control	Logit (Propensity Score)	Distances
Obs43	−2.067	Obs717	−2.067	0.000
Obs91	−0.851	Obs1663	−0.850	0.009
Obs173	−2.033	Obs628	−2.033	0.001
Obs189	−1.626	Obs79	−1.627	0.008
Obs240	−1.265	Obs1601	−1.265	0.002
Obs253	−1.994	Obs287	−1.994	0.000
Obs311	−1.236	Obs1546	−1.238	0.015
Obs353	−0.593	Obs657	−0.596	0.028
Obs394	−0.836	Obs1165	−0.834	0.017
Obs423	−0.563	Obs144	−0.561	0.016
Obs456	−2.036	Obs921	−2.036	0.000
Obs460	−2.004	Obs1478	−2.004	0.000
Obs467	−0.542	Obs1494	−0.539	0.023
Obs519	−1.747	Obs1386	−1.747	0.003
Obs573	−2.068	Obs875	−2.068	0.001
Obs593	−2.080	Obs954	−2.080	0.000
Obs752	−2.054	Obs1403	−2.054	0.000
Obs817	−1.801	Obs382	−1.801	0.002
Obs905	−2.022	Obs1307	−2.022	0.000
Obs1144	−0.210	Obs1177	−0.211	0.007
Obs1149	−0.169	Obs1819	−0.179	0.080
Obs1154	−0.245	Obs269	−0.248	0.017
Obs1171	−0.202	Obs1364	−0.202	0.004
Obs1191	−0.227	Obs1263	−0.225	0.012
Obs1192	−0.198	Obs1189	−0.198	0.000
Obs1228	−0.236	Obs1720	−0.236	0.001
Obs1229	−1.191	Obs1463	−1.191	0.000
Obs1248	−0.184	Obs1688	−0.184	0.007
Obs1401	−2.066	Obs1559	−2.066	0.000
Obs1412	−0.220	Obs1579	−0.220	0.000
Obs1413	−1.376	Obs40	−1.393	0.128
Obs1434	−0.160	Obs1423	−0.158	0.014
Obs1609	−0.061	Obs865	−0.064	0.023
Obs1623	−2.028	Obs1821	−2.028	0.000
Obs1626	−1.275	Obs100	−1.264	0.088
Obs1648	−1.080	Obs1597	−1.085	0.042
Obs1674	−0.206	Obs1832	−0.203	0.024
Obs1698	−0.186	Obs1690	−0.189	0.025
Obs1752	−0.199	Obs1751	−0.199	0.000
Obs1753	−0.228	Obs1255	−0.229	0.010
Obs1793	−0.238	Obs1538	−0.238	0.000
Obs1795	−0.213	Obs1725	−0.214	0.007
Obs1797	−0.186	Obs1812	−0.186	0.003

## Appendix B. List of Variables Being Used for the Classification and Regression Tree (CART) Analysis and Multivariable Ordinal Logistic Regression

**Table A3:** List of variables being used for the Classification and Regression Tree (CART) analysis and multivariable ordinal logistic regression.

Continuous Variables
Age
BMI
Gestational age
Gravidity
Parity
SOFA score
APACHE II
ROx index
Shock Index
WBC
Lactate
AST
ALT
LDH
Uric acid
INR
Categorical variables
Race
Marital status
PMHx—Hypertension
PMhx—DM
PMhx—CKD
PMhx—Asthma
PMHx—cardiac disease
Social history—Intravenous drug use
Social history—Smoking
Psychiatric history—anxiety or depression
Any obstetric history—Postpartum hemorrahge Or Preeclampsia OR multifetal gestation OR Previa/Placenta abruption
Any Gynecologic history—uterine fibroid OR Cerclage OR PNC

## Appendix D

**Table A4:** Results of ordinal logistic regression to measure the association between patient characteristics and time interval between admission to MFW and ICU admission. Upgrade time interval was ranked from 0 (0–12 h), 1 (12.1–24.0 h), and 2 (>24.0 h).

Variables	Coefficient	OR	95% CI	P
Age	0.07	1.07	0.93–1.24	0.34
BMI	−0.02	0.98	0.89–1.09	0.73
Gravidity	−0.28	0.76	0.42–1.35	0.35
Parity	0.11	1.11	0.49–2.54	0.80
Hospital admission SOFA	−0.19	0.83	0.49–1.41	0.49
Hospital admission APACHE-II	0.36	1.44	1.08–1.91	0.01
ICU admission SOFA	−0.04	0.96	0.64–1.42	0.82
ICU admission APACHE-II	−0.08	0.92	0.75–1.13	0.42
Hospital admission Shock Index	−0.73	0.48	0.01–25.57	0.72
Hospital admission WBC	−0.02	0.98	0.76–1.26	0.87
ICU admission WBC	0.05	1.06	0.87–1.29	0.59
Black	0.70	2.01	0.33–12.27	0.45
Married	0.28	1.32	0.25–6.98	0.74
Preeclampsia	−2.96	0.05	0–2.37	0.13
Hypertension	2.65	14.19	1.69–119.38	0.02
Asthma	−0.22	0.8	0.13–5.02	0.82
IVDU	−1.92	0.15	0.01–1.88	0.14
Tobacco	3.16	23.47	1.29–427.3	0.03
Anxiety/Depression	0.04	1.04	0.11–10.22	0.97
Any OB history	0.35	1.42	0.3–6.68	0.66
Any Gynecology history	1.24	3.45	0.4–29.55	0.26
Past medical history of DM	−0.05	0.95	0.09–9.94	0.97
Covid Pos (y/n)	1.00	2.71	0.31–24.01	0.37

Sommer’s D = 0.65; Goodman-Kruskal Gamma = 0.65; Kendall’s Tau-a = 0.43. Abbreviations: BMI, Body Mass Index; SOFA, sequential organ failure assessment; APACHE, Acute Physiology and Chronic Health Evaluation; WBC, white blood cell; CHTN, chronic hypertension; IVDU, intravenous drug use; DM, Diabetes Mellitus.
